# Assessment of Human Exposure Levels Due to Mobile Phone Antennas in 5G Networks

**DOI:** 10.3390/ijerph19031546

**Published:** 2022-01-29

**Authors:** Marta Bonato, Laura Dossi, Silvia Gallucci, Martina Benini, Gabriella Tognola, Marta Parazzini

**Affiliations:** 1 Institute of Electronics, Computer and Telecommunication Engineering (IEIIT), National Research Council, 20133 Milano, Italy; laura.dossi@ieiit.cnr.it (L.D.); silvia.gallucci@ieiit.cnr.it (S.G.); martina.benini@ieiit.cnr.it (M.B.); gabriella.tognola@ieiit.cnr.it (G.T.); marta.parazzini@ieiit.cnr.it (M.P.); 2 Department of Electronics, Information and Bioengineering (DEIB), Politecnico di Milano, 20133 Milano, Italy

**Keywords:** human exposure, mm-wave spectrum, 5G networks, 5G mobile phased-array antenna, deterministic dosimetry

## Abstract

The recent deployment of 5G networks is bringing benefits to the population but it is also raising public concern about human RF-EMF exposure levels. This is particularly relevant considering the next 5G mobile devices, which are placed in close proximity to the subjects. Therefore, the aim of the following paper is focused on expanding the knowledge of the exposure levels in 5G exposure scenarios, specifically for mobile applications, using computational methods. The mobile antenna was designed considering the 5G technology innovations (i.e., mm-wave spectrum, beamforming capability, high gain and wide coverage), resulting in a phased-array antenna with eight elements at the working frequency of 27 GHz. To assess the exposure levels, different types of skin models with different grades of details and layers were considered. Furthermore, not only was the presence of a mobile phone user simulated, but also that of a person in their proximity, who could be hit by the main beam of the phased-array antenna. All the simulations were conducted in Sim4Life platform, where the exposure levels were assessed in terms of absorbed power density averaged over 4 cm^2^ and 1 cm^2^, following the ICNIRP guidelines. The results highlighted that the use of the homogeneous skin model led to the absorbed power density peaks being greatly underestimated, with respect to those obtained in multilayer skin models. Furthermore, interestingly, we found that the exposure levels obtained for the person passing nearby were slightly higher than those experienced by the mobile phone user himself. Finally, using the allowed input power for real mobile applications, all the values remained below the limits indicated by the ICNIRP guidelines.

## 1. Introduction

In the last decades, the use of wireless communication systems is characterizing more and more our daily lives and this trend seems not to stop, indeed the total amount of mobile traffic is expected to increase dramatically in the coming years [1].

In this context, the recent worldwide deployment of 5th generation (5G) networks represents the next big revolution in mobile communication, providing solutions in order to meet the users’ expected needs in terms of data rate increase, low transmission latency and high device density [2].

Indeed, 5G networks represent an evolution and an expansion with respect to existing 4th generation (4G) networks. One of the key points of 5G networks compared to 4G networks is the use of new additional spectrum, in the range between 3 to 100 GHz, in mobile communications, that will facilitate obtaining wider bandwidths [3]. In Europe, each country is now regulating the new frequency ranges that will be involved in the deployment in 5G networks [4]. In our work, we decided to focus on the first licensed frequencies adopted by Italy in the range of mm-wave spectrum, which will be allocated in the range of 26.5–27.5 GHz [4,5].

The introduction of these new mm-wave frequencies will require the installation of massive multiple-input multiple-output (MIMO) base stations and antennas with three-dimensional (3D) beamforming capabilities [6,7,8] in order to cope with the very high path loss experienced at the higher frequencies and obtaining high focalized beams only in the desired direction [9,10].

The consequent innovations to be applied at base station sites and to mobile devices and the actual implementation in the electronical devices are introducing unprecedented new challenges that are far from trivial [11]. Indeed, regarding specifically mobile phones, the antenna design in 5G networks involves the investigation of phased-array antenna models with high gain, wide coverage and with the beam steering function to countermeasure the high path loss caused by the use of the mm-wave spectrum. This represents a difficult challenge: the suitable phased-array antenna models have to fit within a mobile device, which has strict spatial limitations [12,13,14]. 

Furthermore, due to nearby positioning to human subjects, the assessment of human exposure levels due to the presence of mobile terminals have always been a critical issue for wireless communication systems, in order to define the maximum allowed output power to avoid possible health effects.

This topic is further amplified by the deployment of 5G networks and their innovative technologies, which are involving more and more drastic changes regarding the RF-EMF exposure assessment of the population, especially considering mobile devices, which are placed near to human subjects [5]. Until recent years, little attention has been paid to the assessment of human exposure at frequencies above 6 GHz. With 5G networks this has started to change, indeed, we feel the need to evaluate any harmful effects on health caused by exposure in the range of mm-wave radiation [15]. 

For this reason, the most widely worldwide adopted exposure limits to protect human bodies from exposure to mm-wave radiation, indicated by the guidelines of the International Commission on Non-Ionizing Radiation (ICNIRP), were recently revised, to consider the deployment of 5G networks [16]. As it was underlined from the revised guidelines, the use of RF-wave causes a more superficial absorption in the human tissue, limited to only a few mm depth and mostly localized in the most external tissue (i.e., skin and subcutaneous fat) [16,17]. Therefore, the basic restrictions above 6 GHz indicated in the ICNIRP guidelines are described in terms of the density of absorbed power over area (W/m^2^), referred as “absorbed power density” (S_ab_) averaged over a square area of 4 cm^2^ or 1 cm^2^, depending on the frequency range [16].

Despite the definition of the limits and guidelines and the growing interest in this topic, there are still few computational studies on human exposure level assessment estimation at mm-wave frequency ranges considering mobile applications, mostly because the investigation using numerical approaches is still quite challenging due to the high costs of computational simulations [18]. Some literature studies deal with the calculation of the maximum possible radiated power from mobile devices in proximity to a human body to not cause negative effects [19,20]. Efforts have also been made to determine the maximum averaging area for power density that limits the maximum temperature increase to a given threshold for frequencies above 6 GHz for plane wave, dipoles and array antenna [21]. Furthermore, some research involved the use of realistic anatomical models to assess the exposure levels in terms of specific absorption rate and absorbed power density [22,23,24], whereas others focused on multilayer modeling of the skin when exposed to plane-wave to underline how, at high frequencies, a detailed skin model is necessary to correctly estimate the assessment of exposure levels [25,26,27]. 

The proposed study takes place in this context and our aim is to further broaden the knowledge on the assessment of human exposure with computational methods considering the future 5G mobile devices for Italy. 

More in detail, instead of a simple incident plane-wave, the EMF source was described by a general model of mobile antenna, taking into account the technology novelties in 5G mobile networks, i.e., a phased-array antenna with beamforming capability at the working frequency of 27 GHz, i.e., the first licensed mm-wave frequency in Italy for 5G networks [4,5]. The exposure levels in terms of power absorbed density as indicated in the ICNIRP guidelines were then evaluated in three different types of geometrical skin-layers models, to verify how the details in the models could impact on the exposure levels. Additionally, a novelty in the study is to investigate the exposure levels simulating the presence of not only the mobile phone user, but also of a person placed in the proximity of the user’s mobile phone. Details and results on the construction of the antenna, on the skin models and on the different configurations examined are reported in the following paragraphs.

## 2. Materials and Methods

### 2.1. Antenna Model

**Figure 1:**
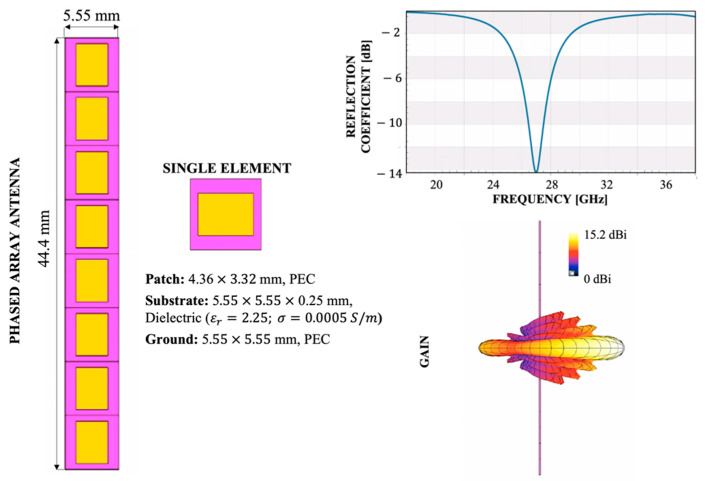
Configuration of the phased-array antenna for mobile phone applications. On the left, the antenna geometry; on the right, the reflection coefficient plot and the antenna gain pattern.

In the generation of 5G mobile devices, to overcome the problem of high propagation loss due to the use of mm-wave spectrum, phased-array antennas with high gain and beamforming capability are becoming a solution, although it is still a challenge to obtain this wide coverage within the narrow space presented in mobile handset [12,13,14]. For this reason, as shown in Figure 1, in the present work the design of the simulated antenna was chosen according to all these features implemented in the next generation of 5G mobile devices. The antenna model and its parameters were implemented using the Sim4Life software platform (www.zmt.swiss (accessed on 21 December 2021)).

As can be seen, the antenna model is indeed characterized by a phased antenna with beamforming capability composed by an array of 8 elements, with dimensions equal to half wavelength (i.e., around 5.55 mm at 27 GHz), resulting in a total array of 44.4 × 5.55 × 0.246 mm, compatible with the area for the space allocation in mobile phones [28,29]. Each single antenna of the phased array is characterized by a patch antenna composed by three layers. In more detail, the ground and patch layers were modelled by perfect electric conductor (PEC) materials and the substrate layer was modeled by a dielectric material (dielectric properties from literature [30]: ε_r_ = 2.25 and σ = 0.0005 S/m). The dimensions of each single patch antenna were chosen accordingly to have a resonance of the antenna at 27 GHz, in line with the first licensed frequency in Italy for 5G networks [4,5].

Finally, the array antenna is driven by a Gaussian signal with a total input power of 30 dBm (1 W), whereas the different array elements have a phase shift set to zero, resulting in a main lobe of the radiation pattern in the boresight direction, i.e., perpendicular to the plane of the antenna, providing the maximum gain, with a value equal to 15.2 dBi in the air, in line with literature studies [28,29]. 

### 2.2. Anatomical Model of the Skin

As reported in the introduction, it is well known that human EMF exposure caused by mm-wave radiation is localized only in the most superficial tissues of humans, i.e., the skin tissue.

Furthermore, for mm-wave frequencies up to 100 GHz, modelling the skin by a single layer of homogeneous dermis tissue with constant dielectric properties over its entire thickness seems an oversimplification, no longer justified because of the shorter wavelength and penetration depth. Consequently, literature studies suggested that a detailed knowledge of the skin structure could be necessary to produce reliable estimations of the absorption of RF radiation and an accurate multilayer model of skin and subcutaneous tissue should be taken into account to evaluate the absorption of RF energy throughout the skin and near-surface tissues [17,25,26,27]. 

The aim of the present work is to further investigate this topic, comparing the absorbed power densities estimated by using three different 3D geometrical models of skin tissue with different numbers of layers. 

The three different 3D models, i.e., homogeneous, three layers and four layers, and the thickness chosen for each layer are reported in Table 1, whereas Table 2 reports the dielectric properties of the involved tissues, based on literature [17,25,31,32].

In more detail, the homogeneous model is the easiest and most simplified one, largely used in literature studies, where the skin is characterized by only one homogeneous single layer, as the name implies. The dielectric properties of the layer are equal to the dermis properties [31,32]. The model has a total dimension of 20 × 20 cm and a depth supposed infinity, in line with the dimension chosen in [22].

The other two multilayer models allow the exposure levels to be assessed more accurately through the stratified characteristics of the skin. Considering the short penetration depth of RF energy into skin at mm-wave frequencies, one model is characterized by a three-layer structure. Starting from the external to the inner layer, we simulated the layers of the stratum corneum, the viable epidermis and dermis, and the fat [17,25]. Finally, the four-layer model introduces a fourth deeper layer, with the dielectric properties of the muscle tissue, to investigate the energy penetration depth at a frequency lower than 30 GHz and how much energy could also be absorbed in the muscle layer [17]. The total dimensions of the two multilayer models are the same as those of the homogeneous model (20 × 20 cm); furthermore, the thicknesses of each layer, as reported in Table 1, are the ones that caused the highest exposure levels, according to the data found in literature [25].

### 2.3. Exposure Assessment

**Figure 2:**
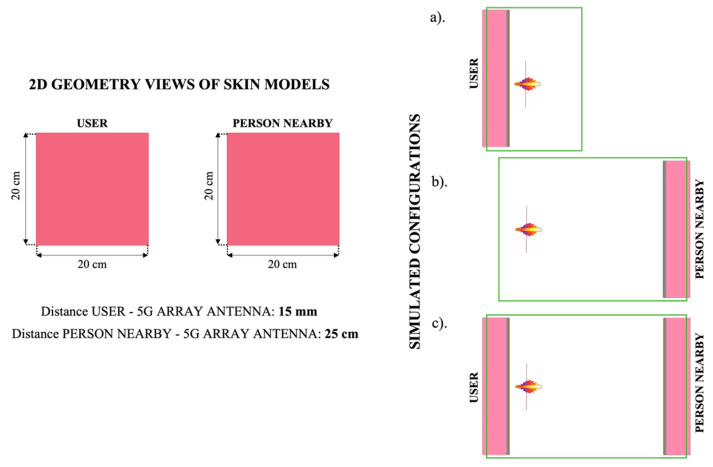
On the left, the 2D skin model geometry views of the mobile phone user and of a person who is passing nearby; the depth of the skin models is supposed as infinity, thanks to the position of the simulation boundary conditions (in green on the right). On the right, the three different configurations are shown with the simulation boundary conditions (in green), designed to investigate: (**a**) the exposure levels of the person using the mobile phone at a distance of 15 mm, alone; (**b**) the exposure levels of a person at a distance of 25 cm from the mobile phone (assuming that only the phone is present, while the mobile user is not present); (**c**) the exposure levels of both the user and a person in his proximity, when they are simultaneously present.

As reported in the introduction, concerning the exposure assessment, the present work estimates the exposure levels not only of a mobile phone user, but also of a person that could be in his proximity, thus, also exposed to the radiation of the mobile phone antenna. In Figure 2, there are illustrated the 2D geometry views of the user and the person nearby, the considered distances between the models and the 5G array antenna and the three different exposure assessment configurations that were simulated for each skin model.

The first configuration has been designed to evaluate the exposure levels of only the person who is using the mobile phone. In this case, the distance between the mobile antenna and the skin model is set to be equal to 15 mm.

In the second configuration, we investigate the exposure levels of a person in the proximity of the mobile phone, at a distance of 25 cm, assuming that only the phone, but not the user, is present.

Finally, the third configuration implies the simultaneous presence of the user and the person in his proximity. The comparison between the exposure levels obtained for the user and the neighbor in the three different configurations will help us to better interpret the results. 

All the simulations were performed using the Sim4Life platform (www.zmt.swiss (accessed on 21 December 2021)), selecting the finite different time domain method (FDTD), which permits a direct solution of Maxwell’s curl equations to be obtained in the time domain [33]. The computational domain was discretized with a nonuniform grid automatically created by Sim4Life software for the antenna and the surrounding of the models with a subwavelength resolution of around 15 samples per 27 GHz wavelength. We manually selected the grid for the three different skin models with a maximum step varying from 0.26 mm to 0.22 mm depending on the dielectric properties of the model, in order to correctly discretize all the tissues. Moreover, the computational domain was truncated by assuming a perfectly matched layer (PML) absorbing condition at the domain boundaries [34].

To assess the exposure levels, for each tissue presented in the three skin models the maximum absorbed power density values S_ab_ [W/m^2^] were calculated, averaged over 4 cm^2^ and over 1 cm^2^, since the selected frequency of 27 GHz is close to 30 GHz, where focal beam exposure can occur. Indeed, in the ICNIRP guidelines an additional spatial average of 1 cm^2^ is used to ensure that the operational adverse health effect thresholds are not exceeded over smaller regions [16].

## 3. Results

Here, the results of the exposure levels obtained for the three skin models in the different configurations are reported in terms of absorbed power density, as indicated in the ICNIRP guidelines [16]. For simplicity, all the figures and values here reported are evaluated considering at the array antenna a total input power of 30 dBm (1 W), as described in the Materials and Methods paragraph. 

**Figure 3:**
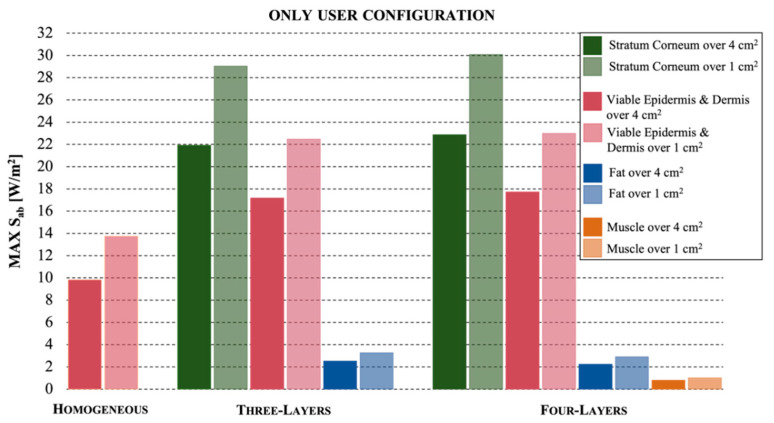
The maximum values of absorbed power density for the configuration where the user is using the mobile phone with a distance of 15 mm, for the three different skin models.

Figure 3 refers to the configuration where the skin model of the user was at a distance of 15 mm from the antenna and there was no neighbor.

The maximum values of the absorbed power density S_ab_ are reported for the different tissues in each skin model. The comparison between the responses of the models highlights that the homogeneous one greatly underestimates the maximum exposure levels measured for the multilayer ones. In fact, the values S_ab_ = 9.8 W/m^2^ and 13.8 W/m^2^ estimated with the homogeneous model, over an area of 4 cm^2^ and 1 cm^2^, respectively, are reduced of about 54% with respect to the highest exposure levels achieved in the stratum corneum of the multilayer models, that show values S_ab_ = 22.9 W/m^2^ and 30.1 W/m^2^ in the four-layer model and S_ab_ = 21.9 W/m^2^ and 29 W/m^2^ in the three-layer model, always over 4 cm^2^ and 1 cm^2^, respectively. The viable epidermis and dermis layer showed similar values in the multilayer models, i.e., 17.7 W/m^2^ over 4 cm^2^ and 23 W/m^2^ over 1 cm^2^ for three layers and 17.2 W/m^2^ and 22.5 W/m^2^ for four layers. The fat tissue also showed similar results in the multilayer models, with very low values reduced by about 90% with respect to the maximum values, i.e., 2.3 W/m^2^ over 4 cm^2^ and 2.9 W/m^2^ over 1 cm^2^ for three layers and 2.5 W/m^2^ and 3.3 W/m^2^ over 1 cm^2^ for four layers. Lastly, the muscle, the fourth tissue in the four-layer model, showed negligible exposure values reduced by 97% with respect to maximum values.

**Figure 4:**
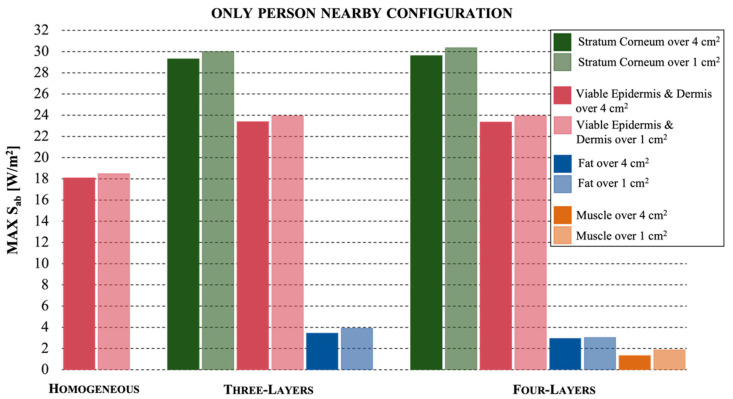
The maximum values of absorbed power density for the configuration where there is a person in the proximity of the mobile phone with a distance of 25 cm, for the three different skin models.

The results shown in Figure 4 refer to the configuration that simulated the presence of a person at 25 cm from the antenna of the mobile phone, in the absence of the user.

Although the distance between the skin models and the antenna was largely greater with respect to the first configuration, the results showed similar higher peaks, again for the stratum corneum in the multilayer models. It can be noticed that the S_ab_ values on the area of 4 cm^2^ were similar to those measured on 1 cm^2^, differently from Figure 3. Similar to the first configuration, instead, we found that (i) the homogeneous tissue model showed a reduction of 39% on the assessment of the exposure levels (absorbed power density peaks of 18 W/m^2^ over 4 cm^2^ and 18.5 W/m^2^ over 1 cm^2^), that suggests that the homogeneous model does not provide an accurate estimation; (ii) the three-layer and the four-layer models seemed to behave in the same way; the fourth layer of muscle tissue showed negligible contribution in the evaluation of exposure (only 0.01% of the highest measured value); (iii) the stratum corneum, averaged either on 4 cm^2^ or 1 cm^2^, and for both multilayer models, showed values around 30 W/m^2^; (iv) in dermis tissue the values decreased to about 25 W/m^2^ and (v) the exposure levels in fat tissue showed very low results, with a reduction of 90% with respect to the highest value simulated in the stratum corneum tissue.

**Figure 5:**
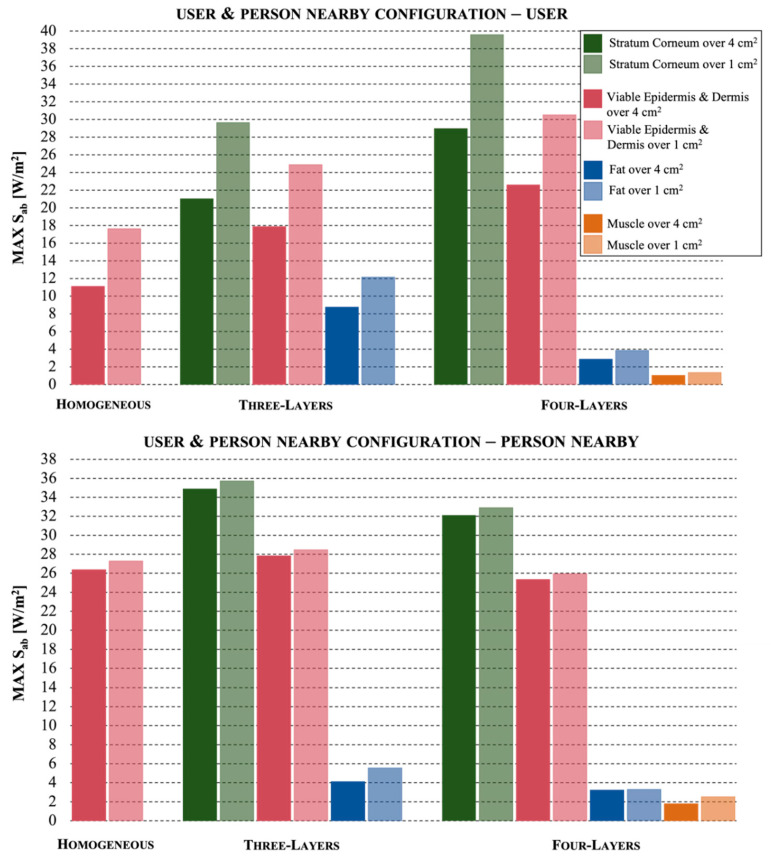
The maximum values of absorbed power density for the configuration where there is the simultaneous presence of the user and of a person in proximity to the user, for the three different skin models. In the upper part, the values obtained for the user and in the lower part the values obtained for the person nearby.

Finally, Figure 5 reports the results obtained in the configuration when both the user and the person near the mobile phone were simulated: in the upper part, the Figure shows the exposure levels in the skin models of the user, whereas in the lower part, those of the person nearby. At first glance, we notice that here the exposure values were slightly higher with respect to the results obtained in the previous configurations, where only the user or the neighbor were present.

First of all, as expected in this configuration, the homogeneous skin model also underestimated the exposure levels, with a significant reduction up to 55% for the user (with peak value equal to 11.1 W/m^2^ over 4 cm^2^ and 17.6 W/m^2^ over 1 cm^2^) and a moderate reduction of 18% for the person in the proximity to the antenna (with peak value equal to 26.4 W/m^2^ over 4 cm^2^ and 27.3 W/m^2^ over 1 cm^2^) with respect to the maximum values in the stratum corneum. 

Comparing the exposure level results in the skin models of the user in the configuration where he is alone (Figure 3) and in the presence of a neighbor (Figure 5, upper part), we highlight some differences. The fat tissue in the three-layer model, but not in the four-layer model, showed increased exposure levels, 8.8 W/m^2^ over 4 cm^2^ and 12.2 W/m^2^ over 1 cm^2^. Moreover, the stratum corneum in the four-layer, but not in the three-layer model, showed a significant increase, achieving 29 W/m^2^ over 4 cm^2^ and 39.6 over 1 cm^2^ for the stratum corneum, with an increment up to 32% over 1 cm^2^. This means that when a person is in the proximity of the mobile user, the user multilayer skin model has a different behavior.

Comparing the exposure level results in the skin models of the person at 25 cm from the mobile antenna in the configuration where he is alone (Figure 4) and in the presence of the user (Figure 5, lower part) we highlight some differences. While the values obtained for the four-layer model are almost the same, with peak values equal to 32.1 W/m^2^ over 4 cm^2^ and 32.9 W/m^2^ over 1 cm^2^ for the stratum corneum, significant increases are registered for the exposure levels of the homogeneous model, increased from about 17 W/m^2^ to about 27 W/m^2^ and for the tissues of the three-layer skin model, with peak exposure values of 34.9 W/m^2^ over 4 cm^2^ and 35.7 W/m^2^ over 1 cm^2^ for the stratum corneum (with an increment of 18% with respect to the values evaluated in the absence of the mobile user).

It is confirmed, for all the configurations, that only the most superficial layers of the multilayer models (stratum corneum and viable epidermis and dermis, with values equal to 25.3 W/m^2^ over 4 cm^2^ and 25.9 W/m^2^ over 1 cm^2^) showed high values, whereas the fat and muscle layers had really low values (a reduction in the exposure levels up to 90% and 97%, respectively, with peak values equal to 3.2 W/m^2^ over 4 cm^2^ and 3.3 W/m^2^ over 1 cm^2^ for the fat and 1.8 W/m^2^ over 4 cm^2^ and 2.5 W/m^2^ over 1 cm^2^ for the muscle). 

## 4. Discussion

Our aim was to expand the knowledge about the exposure level assessment considering the novelty of 5G mobile devices. To obtain this, we modelled the device antenna by a phased-array antenna at 27 GHz, the new allocated range of frequencies for 5G networks [12,13,14]. Since it is known that the exposure at mm-wave radiation affects only the first millimeters of depth of human tissue, different types of skin layer models were taken into account to allow us to highlight the most accurate skin model for investigating the radiation absorption of the different layers of tissue.

As expected, the results showed that the radiation focused only on the superficial part of the skin models, specifically in the stratum corneum and in the viable epidermis and dermis tissues. Indeed, the deeper layers (i.e., fat and muscle tissues) showed a reduction of the peak absorbed power density values up to 90% and 97%, respectively, with respect to more superficial tissues. This is in line with other literature studies, which estimated that the energy penetration depth in tissue becomes very small at mm-wave frequencies (<1 mm above about 15 GHz) and consequently the energy is rapidly absorbed within the stratum corneum, the viable epidermis and the dermis and does not reach the deeper tissues in the body [17,18,25,26,27]. 

Furthermore, because of this small penetration depth, it was evident that a detailed model of the human skin tissue was then necessary at mm-wave spectrum, to correctly assess the exposure levels due to the multiple reflections of electromagnetic waves that can occur at the boundaries between the different tissue layers with different dielectric properties. Indeed, the results confirmed that a homogenous skin model is not appropriate for estimating the absorbed power density, underestimating the exposure by 18% to 55% with respect to the maximum values registered in multilayer models. This is again in line with the study of Chirst et al. [25], where it was underlined that modelling the skin as homogeneous dermis tissue could underestimate the induced temperature increase by more than a factor of three. 

Our simulations showed that the differences between the results obtained for the three-layer model and the four-layer model were instead not very relevant in assessing the exposure levels. Indeed, in general, both the models seemed adequate, with maximum differences on the peak exposure levels around 8% for the stratum corneum layer in all the cases, with the only exception for the user’s exposure levels in the configuration where both the user and the person nearby were simulated; in this specific case the difference in the peak values between the two models was up to 27.6%. This consideration is in line with literature studies where it was specified that for frequencies of 15 GHz and above, the impact of the muscle layer is significantly diminished, due to both the generally lower depth of penetration at higher frequencies, and to the increasing electrical conductivity in the fat tissue and for this reason for frequencies above 30 GHz, a simple three-layer model is sufficient [17,25].

Entering into more detail on the numerical results for each investigated configuration, the user absorbed power density peaks obtained for the homogenous skin model were in line with the work of Morelli et al. [24], where the exposure levels caused by a microstrip patch antenna array (at 28 GHz) for mobile applications were evaluated in different human models with a single skin layer. The peak values of the different human models were indeed in the range between 0.13–0.43 W/m^2^ for an input power of 15 dBm (32 mW). In the present paper, rescaling the values for the same input power, we obtained 0.31 W/m^2^ for the homogeneous skin, and higher values, i.e., 0.7 W/m^2^ and 0.73 W/m^2^, respectively, for the three-layer and four-layer models. This is a further confirmation of the above considerations about the necessity to use a multilayer model in the mm-wave spectrum so as not to underestimate the total exposure.

Moreover, comparing the peak of absorbed power density for the configurations where the user and the person nearby are simulated separately, we found, interestingly, that the person in proximity of the mobile phone user had almost the same (or slightly higher) exposure levels as the user himself, despite the distances between the mobile phone being greatly different (15 mm for the user vs. 25 cm for the person nearby). This is probably because, although in a position further away from the array antenna, the person nearby is hit by the main lobe of the antenna (see Figure 1) while the user is hit by the back lobe of the antenna pattern. This is in line with the results of Thors at al. [11], that showed that for large array antennas the maximum exposure may occur some distance away from the face of the array. This suggests that in the future, to guarantee a safe environment, it will be fundamental to evaluate the exposure levels not only of the mobile phone users but also of people nearby that could be hit by the primary beam of the antenna array. 

Considering the practical configuration, where both the user and the person nearby are present, we noticed an increment of the exposure levels, up to 32% for the user and up to 18% for the person nearby, suggesting that future efforts should also be focused on evaluating how the reciprocal positions between mobile phone users and their possible neighbors could influence the total exposure levels.

Lastly, with the reference power input of 30 dBm (1 W) used in the simulations, the absorbed power density peaks in the stratum corneum layer in all the configurations were over the value of 20 W/m^2^, indicated as the limit in the ICNIRP guidelines to avoid harmful effects. However, it is important to underline that the maximum input power of the phased-array antenna in real mobile applications will be around 23 dBm (200 mW), as indicated by the specifications of 3rd Generation Partnership Project and literature studies [3,29,35]. The use of these input powers will greatly reduce, by a factor of five, the simulated values of exposure so complying with the indicated ICNIRP guidelines limits.

## 5. Conclusions

In conclusion, the aim of further expanding the knowledge about 5G mm-wave exposure in mobile applications was achieved. The results confirmed that at these high frequencies it is essential to consider a multilayer model of the skin rather than a simple homogeneous one that could lead to greatly underestimating the exposure levels. Furthermore, the study highlights those efforts should be focused not only on evaluating the exposure levels for the mobile phone users but also for people passing nearby, who could be hit by the main lobe of the mobile antenna pattern. Finally, it is important to underline that the maximum values of S_ab_ obtained in all the conducted simulations, when scaled for a real input power (23 dBm, i.e., 200 mW) respect the basic restrictions indicated in the ICNIRP guidelines to avoid harmful effects. Future work will deal with the investigation of detailed skin tissues to be used in anatomical models, to take into account the real morphology of human subjects, and the study of the impact of the beamforming capability of the mobile antenna on the exposure scenario.

## Figures and Tables

**Table 1:** Thickness of the layers in the three different skin models.

Tissue	Homogeneous	Three Layers	Four Layers
Stratum corneum	/	0.7 mm	0.7 mm
Viable epidermis and dermis	∞	0.96 mm	0.96 mm
Fat	/	∞	1.6 mm
Muscle	/	/	∞

**Table 2:** Dielectric properties of the different skin layers.

Tissue	Relative Permittivity	Conductivity [S/m]	Mass Density [kg/m^3^]
Stratum corneum	3.52	1.21	1500
Viable epidermis and dermis	17.12	25.13	1109
Fat	3.73	1.65	911
Muscle	25.13	32.62	1090

## Data Availability

Not applicable.
